# Enhanced antifungal activity of posaconazole against *Candida auris* by HIV protease inhibitors, atazanavir and saquinavir

**DOI:** 10.1038/s41598-024-52012-8

**Published:** 2024-01-18

**Authors:** Yehia Elgammal, Ehab A. Salama, Mohamed N. Seleem

**Affiliations:** 1 Department of Biomedical Sciences and Pathobiology, Virginia-Maryland College of Veterinary Medicine, Virginia Polytechnic Institute and State University, 1410 Prices Fork Rd, Blacksburg, VA 24061 USA; 2 Center for One Health Research, Virginia Polytechnic Institute and State University, Blacksburg, VA 24061 USA

**Keywords:** Antifungal agents, Biofilms

## Abstract

The increasing incidence and dissemination of multidrug-resistant *Candida auris* represents a serious global threat. The emergence of pan-resistant *C. auris* exhibiting resistance to all three classes of antifungals magnifies the need for novel therapeutic interventions. We identified that two HIV protease inhibitors, atazanavir and saquinavir, in combination with posaconazole exhibited potent activity against *C. auris *in vitro and in vivo*.* Both atazanavir and saquinavir exhibited a remarkable synergistic activity with posaconazole against all tested *C. auris* isolates and other medically important *Candida* species. In a time-kill assay, both drugs restored the fungistatic activity of posaconazole, resulting in reduction of 5 and 5.6 log_10_, respectively. Furthermore, in contrast to the individual drugs, the two combinations effectively inhibited the biofilm formation of *C. auris* by 66.2 and 81.2%, respectively. Finally, the efficacy of the two combinations were tested in a mouse model of *C. auris* infection. The atazanavir/posaconazole and saquinavir/posaconazole combinations significantly reduced the *C. auris* burden in mice kidneys by 2.04- (99.1%) and 1.44-log_10_ (96.4%) colony forming unit, respectively. Altogether, these results suggest that the combination of posaconazole with the HIV protease inhibitors warrants further investigation as a new therapeutic regimen for the treatment of *C. auris* infections.

## Introduction

Invasive fungal infections pose an underestimated threat to the public health^1,2^. Fungal infections are on the rise globally, particularly among immunocompromised individuals, reaching over 11 million infections and 1.5 million deaths annually^3–5^. The majority of invasive fungal infections is caused by *Candida* species which are associated with high mortality rates (up to 60%)^2,6^. The emerging fungal species, *Candida auris* has spread to more than 45 countries, causing serious hospital outbreaks, and significantly contributed to the worldwide health challenge of antifungal resistance^7^. The incidence and dissemination of multidrug-resistant *C. auris*-related infections are alarmingly growing and are associated with high mortality rates^8^. Thus, there is a desperate need to develop new antifungals for the treatment of multidrug-resistant *C. auris* infections.

*Candida* *auris* possesses an arsenal of virulence factors known to contribute to its pathogenicity. Among these, biofilm formation is a major virulence factor of *C. auris* which plays an important role in the nosocomial outbreaks of life-threatening invasive candidiasis^9^. Notably, *C. auris* infections are frequently associated with medical devices such as catheters, where it forms biofilms that adhere to the device surface, leading to device malfunction and serving as a source of bloodstream infection^10^. Furthermore, the formation of biofilms significantly contributes to the escalation of antifungal resistance, as these biofilms enable *C. auris* cells to tolerate higher concentrations of antifungal agents^10,11^. Consequently, novel therapeutic approaches are urgently needed to effectively manage *C. auris* infections, particularly those associated with biofilm formation^9^.

Posaconazole, a second-generation azole antifungal, has a potent and broad-spectrum antifungal activity against pathogenic fungi, including *C. auris*^12^. Posaconazole has a more favorable safety and tolerability profile when compared to the other azoles like fluconazole, itraconazole, and voriconazole. Long-term treatment with posaconazole has demonstrated a reduced risk of inducing side effects^13,14^. Furthermore, posaconazole’s extended half-life of 15–35 h, widespread tissue distribution, and gradual elimination contribute to its therapeutic effectiveness^15^. In addition to these attributes, posaconazole offers therapeutic advantages over fluconazole and voriconazole because it is less likely to be affected by mutations in the ergosterol gene (*ERG11*), which codes for lanosterol demethylase, the target enzyme of azoles^11^. Furthermore, it was reported that patients infected with fluconazole-resistant *C. glabrata* or *C. tropicalis* were able to recover when treated with posaconazole^1^. However, the emergence of pan-resistant *C. auris*, displaying resistance to all three classes of antifungals, underscores the urgency for the development of new therapeutics^16^.

Previously, we reported that lopinavir exhibited potent synergistic interactions with fluconazole, itraconazole and voriconazole against *C. auris*^17^. We also identified atazanavir and saquinavir as potent drugs that restored the activity of itraconazole against *C. auris *in vitro and in vivo^18,19^. Building upon our previous studies, we aimed herein to enhance the activity of posaconazole against *C. auris* using the HIV protease inhibitors (atazanavir and saquinavir). We also evaluated the activity of the combinations of posaconazole with atazanavir and saquinavir in inhibiting the *C. auris* biofilm. Finally, we investigated the in vivo efficacy of atazanavir/posaconazole and saquinavir/posaconazole combinations in a mouse model of *C. auris* infection*.*

## Results

### Posaconazole exhibits synergistic antifungal activity with atazanavir and saquinavir in vitro

Checkerboard assay was performed to corroborate the potential synergistic interactions for the combinations atazanavir/posaconazole and saquinavir/posaconazole against 17 *C.* *auris* isolates. Atazanavir and saquinavir were found to interact synergistically with posaconazole against 100% of *C. auris* isolates decreasing the minimum inhibitory concentrations (MICs) of posaconazole by 4–17 folds. Atazanavir/posaconazole and saquinavir/posaconazole combinations demonstrated synergistic fractional inhibitory concentration indices (FICIs) of (0.06–0.28) (Table 1). Additionally, these two combinations showed similar synergistic interactions against other medically important *Candida* species such as *C. albicans*, *C. tropicalis*, *C. parapsilosis*, *C. krusei* and *C. glabrata* with FICIs ranging between 0.12 and 0.38 (Table 2).

**Table 1:** In vitro synergistic interactions of posaconazole with atazanavir and saquinavir against *Candida auris.*

*C. auris* Isolate	Clade	POS/ATV combination				POS/SQV combination			
		MIC (µg/ml)		ΣFICI	Mode	MIC (µg/ml)		ΣFICI	Mode
		Alone	Combined			Alone	Combined		
CBS 10913	II	0.125/ > 128	0.03/4	0.26	SYN	0.125/ > 128	0.007/8	0.09	SYN
CBS 12372	II	1/ > 128	0.125/16	0.19	SYN	1/ > 128	0.125/4	0.14	SYN
CBS 12373	II	1/ > 128	0.125/16	0.19	SYN	1/ > 128	0.125/4	0.14	SYN
CBS 12766	I	0.5/ > 128	0.125/8	0.28	SYN	0.5/ > 128	0.125/2	0.26	SYN
CBS 12768	I	0.5/ > 128	0.125/8	0.28	SYN	0.5/ > 128	0.125/2	0.26	SYN
CBS 12770	I	1/ > 128	0.125/8	0.16	SYN	1/ > 128	0.125/4	0.14	SYN
CBS 12771	I	0.5/ > 128	0.125/4	0.27	SYN	0.5/ > 128	0.125/4	0.27	SYN
CBS 12772	I	0.5/ > 128	0.125/8	0.28	SYN	0.5/ > 128	0.125/4	0.27	SYN
CBS 12773	I	0.5/ > 128	0.125/4	0.27	SYN	0.5/ > 128	0.125/2	0.26	SYN
CBS 12774	I	0.5/ > 128	0.125/4	0.27	SYN	0.5/ > 128	0.125/4	0.27	SYN
AR0390	I	1/ > 128	0.125/8	0.16	SYN	1/ > 128	0.125/4	0.14	SYN
AR 1100	–	0.125/ > 128	0.007/2	0.06	SYN	0.125/ > 128	0.007/1	0.06	SYN
AR 1101	II	0.5/ > 128	0.125/8	0.28	SYN	0.5/ > 128	0.125/8	0.28	SYN
AR 1102	III	0.5/ > 128	0.125/4	0.27	SYN	0.5/ > 128	0.125/2	0.26	SYN
AR 1103	III	1/ > 128	0.125/8	0.16	SYN	1/ > 128	0.06/16	0.12	SYN
AR 1104	IV	1/ > 128	0.25/8	0.28	SYN	1/ > 128	0.125/4	0.27	SYN
AR 1105	–	0.25/ > 128	0.06/8	0.27	SYN	0.25/ > 128	0.03/4	0.14	SYN

*POS* posaconazole, *ATV* atazanavir, *SQV* saquinavir, *ΣFICI* fractional inhibitory concentration index, *SYN* synergism.

**Table 2:** In vitro synergistic interactions of posaconazole with atazanavir and saquinavir against different *Candida* species.

*Candida* Isolate	POS/ATV combination				POS/SQV combination			
	MIC (µg/ml)		ΣFICI	Mode	MIC (µg/ml)		ΣFICI	Mode
	Alone	Combined			Alone	Combined		
*Candida albicans* NR-29448	0.125/ > 128	0.03/2	0.25	SYN	0.125/ > 128	0.03/1	0.24	SYN
*Candida albicans* ATCC 26790	0.25/ > 128	0.03/8	0.15	SYN	0.25/ > 128	0.03/1	0.12	SYN
*Candida albicans* ATCC 64124	0.125/ > 128	0.03/2	0.25	SYN	0.125/ > 128	0.03/1	0.24	SYN
*Candida tropicalis* ATCC 1369	0.25/ > 128	0.06/16	0.30	SYN	0.25/ > 128	0.03/8	0.15	SYN
*Candida tropicalis* ATCC 13803	0.5/ > 128	0.06/8	0.15	SYN	0.5/ > 128	0.06/2	0.13	SYN
*Candida* *parapsilosis* ATCC 22019	0.5/ > 128	0.125/32	0.38	SYN	0.5/ > 128	0.06/8	0.15	SYN
*Candida krusei* ATCC14243	0.5/ > 128	0.06/16	0.18	SYN	0.5/ > 128	0.06/16	0.18	SYN
*Candida krusei* ATCC 34135	1/ > 128	0.25/4	0.27	SYN	1/ > 128	0.25/2	0.26	SYN
*Candida glabrata* AR0325	4/ > 128	1/32	0.38	SYN	4/ > 128	1/32	0.38	SYN

*POS* posaconazole, *ATV* atazanavir, *SQV* saquinavir, *ΣFICI* fractional inhibitory concentration index, *SYN* synergism.

### Atazanavir and saquinavir enhance the fungistatic activity of posaconazole against *C. auris*

**Figure 1:**
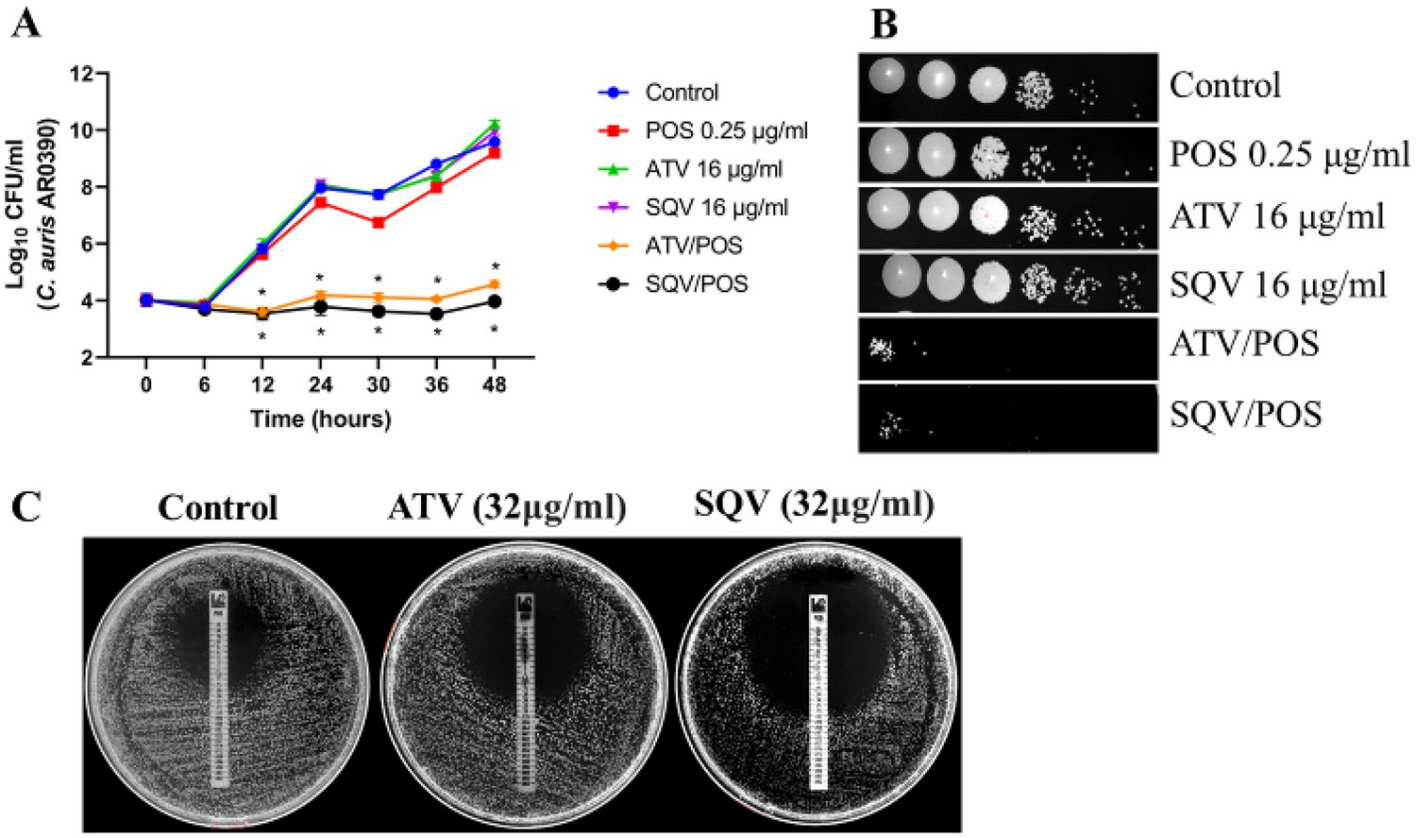
Time-kill of the HIV protease inhibitors (atazanavir (ATV), saquinavir (SQV)), and posaconazole (POS) alone and in combination against *C. auris*. (**A**) Time kill curve of *C*. *auris* AR0390. Data are presented as average log_10_ CFU/ml of *C*. *auris* AR0390 at the corresponding time points. Statistical difference was measured via two-way analysis of variance (ANOVA). An asterisk (*) denotes a statistically significant difference (*P* < 0.0001) from the POS-treated cells. (**B**) Spotting assay was used to visualize the growth kinetics of *C. auris* in the presence and absence of treatments at the 24-h time point. (**C**) Effects of atazanavir and saquinavir on azole-resistant *C. auris*. MIC test strips of posaconazole (ranged from 32–0.002 µg/ml) in the presence and absence of atazanavir and saquinavir at 32 µg/mL.

To investigate the impact of the two combinations on the growth kinetic of *C. auris*, a time-dependent killing assay was conducted against *C. auris* AR0390 for 48 h (Fig. 1). Single drug treatment of either atazanavir (16 µg/ml), saquinavir (16 µg/ml), or posaconazole (0.25 µg/ml) did not affect the fungal growth over time, where their growth pattern was similar to that of the mock (negative) control. However, the atazanavir/posaconazole combination reduced *C. auris* colony forming unit (CFU) by 3.78- and 5.02-log_10_ as compared to the mock (negative) control, after 24 and 48 h, respectively. On the other hand, saquinavir/posaconazole combination reduced *C. auris* CFU by 4.19- and 5.62-log_10_ as compared to the mock (negative) control, after 24 and 48 h, respectively (Fig. 1A). This was confirmed by the spotting assay which showed that *C. auris* had exponential growth in the presence and absence of atazanavir (16 µg/ml), saquinavir (16 µg/ml), posaconazole (0.25 µg/ml). Conversely, the growth of *C. auris* was inhibited significantly in the presence of the two combinations (Fig. 1B). Additionally, we used a posaconazole test strip in RPMI 1640 agar cultured with 10^6^ *C. auris* cells to determine whether HIV protease inhibitors (atazanavir, and saquinavir) improve the activity of posaconazole against azole-resistant *C. auris*. As expected, atazanavir and saquinavir (32 µg/ml) decreased the MIC of posaconazole from 1 µg/ml to 0.38 and 0.19 µg/ml (2.6- and 5.2-folds reduction), respectively, against *C. auris* (Fig. 1C).

### Atazanavir/posaconazole and saquinavir/posaconazole combinations inhibit biofilm formation of *C. auris*

**Figure 2:**
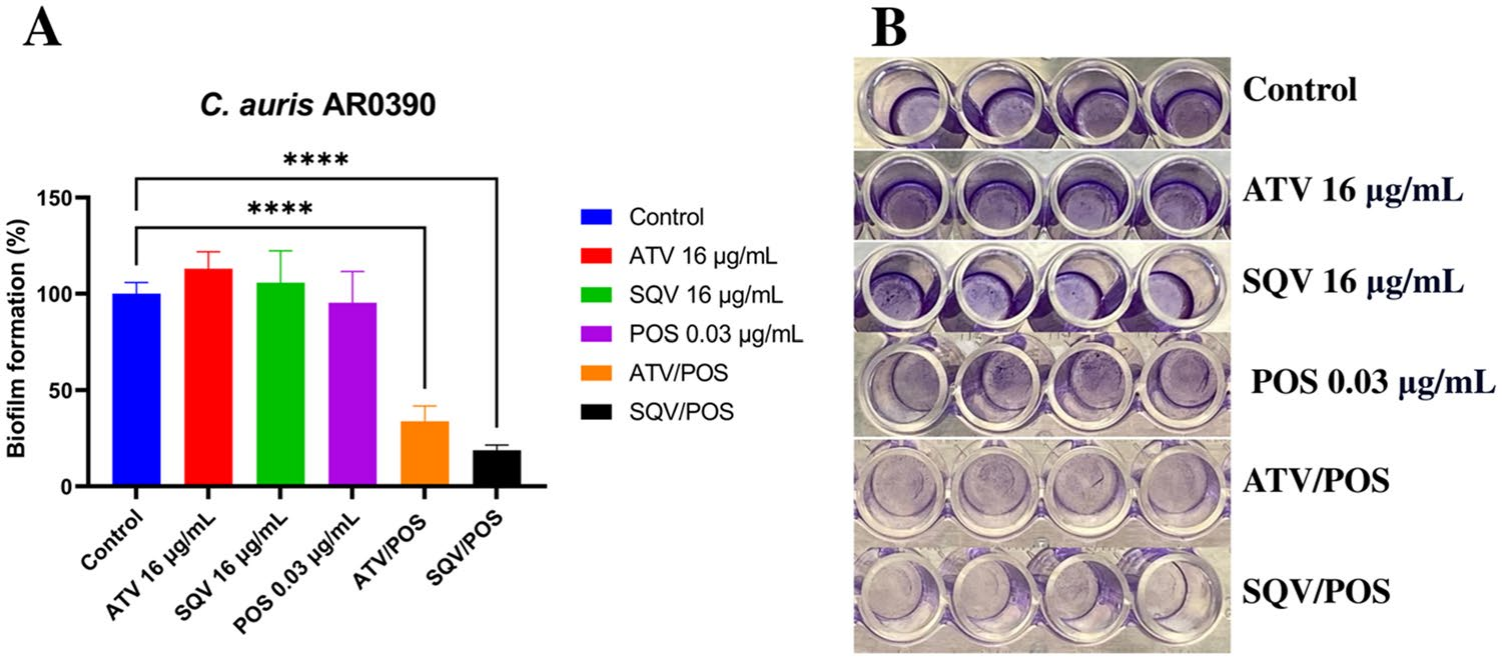
Anti-biofilm activity of the HIV-protease inhibitors/posaconazole combinations. The inhibitory effect of atazanavir/posaconazole (ATV/POS) (16/0.03 µg/mL), and saquinavir/ posaconazole (SQV/POS) (16/0.03 µg/mL) combinations on the formation of *C. auris* AR0390 biofilms was determined using OD_600_ (**A**), which can be visualized using crystal violet (**B**). Statistical difference was measured via One-way Analysis of Variance (ANOVA) with the post hoc Dunnett’s test for multiple comparisons. An asterisk (*) denotes a statistically significant difference (*P* < 0.0001) from the DMSO-treated (control).

Due to the strong antifungal activity against *C. auris*, the two combinations were selected to investigate the biofilm inhibition activity. As shown in Fig. 2, atazanavir, and saquinavir, at a low concentration of 16 µg/ml, in combination with sub-inhibitory concentration of posaconazole (0.03 µg/ml) showed a significant inhibition of the *C. auris* biofilm formation by 66.2%, and 81.2%, respectively, compared to the mock (negative) control.

### Atazanavir and saquinavir enhance posaconazole’s efficacy in a mouse model of *C. auris* infection

**Figure 3:**
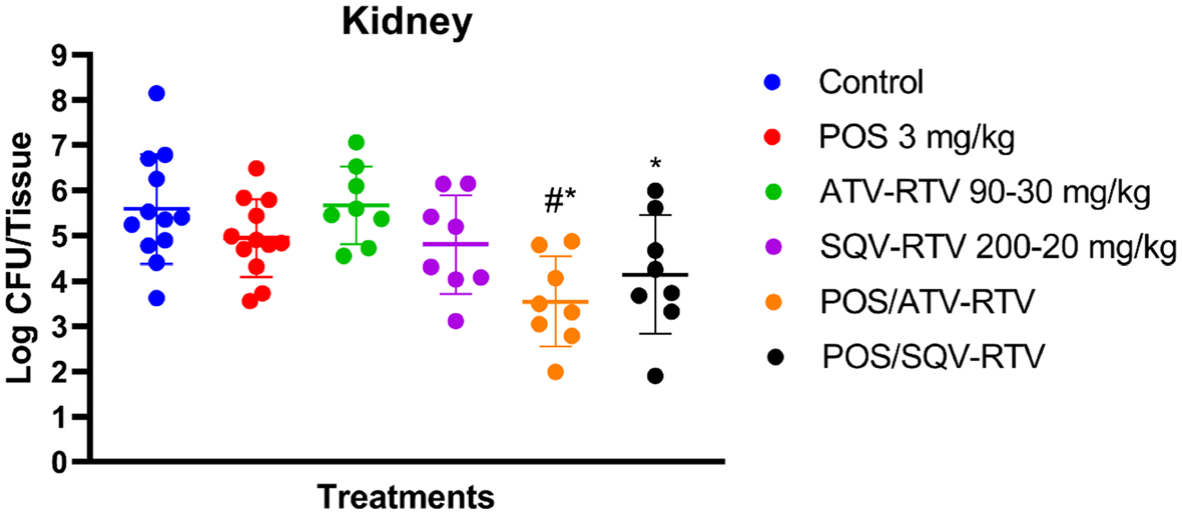
Reduction in fungal load in mice kidneys at 48 h post-infection with *C. auris* AR0390. Treatments were administered orally: posaconazole (3 mg/kg), atazanavir-ritonavir (ATV-RTV; 90–30 mg/kg), saquinavir-ritonavir (SQV-RTV; 200–20 mg/kg), posaconazole/atazanavir-ritonavir (POS/ATV-RTV; 3/90-30 mg/kg), posaconazole/saquinavir-ritonavir (POS/SQV-RTV; 3/200-20 mg/kg) for 2 days. Animals were humanely euthanized after 48 h, and the fungal burden was determined. The data are presented as average CFU in mice kidneys. The data were analyzed using a one-way ANOVA with post-hoc Dunnett's test. An asterisk (*) denotes a statistically significant difference of mice treated with the corresponding treatments as compared to the vehicle-treated mice (*P* < 0.05). A pound (#) indicates a statistically significant difference of mice treated with POS/ATV-RTV combination as compared to POS alone (*P* < 0.05).

A mouse model of disseminated *C. auris* infection was used to investigate the antifungal activity of the two combinations (atazanavir/posaconazole, saquinavir/posaconazole) in vivo. First, the posaconazole dose was optimized to determine the highest dose that does not result in significant reduction in the kidney fungal burden. Mice were treated with different doses of posaconazole (0.25, 0.5, 1, 3, 5 mg/kg) and the optimal dose of posaconazole was determined as 3 mg/kg, which did not result in significant difference as compared to the untreated control (Fig. S1). Next, we tested the in vivo efficacy of the two combinations on this mouse model in the presence of the bioavailability enhancer, ritonavir (to mimic the 3:1 atazanavir: ritonavir and the 10:1 saquinavir: ritonavir ratio used clinically)^20^. The doses chosen of atazanavir/ritonavir (90/30 mg/kg) and saquinavir/ritonavir (200/20 mg/kg) was equal or lower than the dose required in mice to achieve plasma concentration, which is equivalent to the therapeutic levels in humans^21,22^. Treatments were administered orally for 2 days. The next day, mice were euthanized and the *C. auris* burden in their kidneys was determined. Neither posaconazole alone, nor the HIV protease inhibitors (atazanavir–ritonavir, or saquinavir–ritonavir) treatments was capable of reducing the fungal burden of *C. auris* when compared to the untreated control. In contrast, compared to the vehicle control, the posaconazole/atazanavir-ritonavir and posaconazole/saquinavir-ritonavir combinations significantly reduced the burden of *C. auris* in the murine kidneys, producing 2.04 (99.1%) and 1.44-log_10_ CFU (96.4%) reduction. Moreover, when compared to posaconazole treatment, posaconazole/atazanavir-ritonavir and posaconazole/saquinavir-ritonavir combinations generated 1.40 (96%) and 0.8-log_10_ CFU (84.2%) reduction, respectively (Fig. 3).

## Discussion

*Candida* *auris* is a widespread pathogenic yeast causing severe invasive candidiasis in critically ill and immunocompromised patients^23^. Due to its multidrug resistance, severity of infections, and nosocomial outbreaks with high mortality rate, *C. auris* represent an alarming paradigm shift for *Candida* infections^24^. Moreover, the Centers for Disease Control and Prevention (CDC) has classified *C. auris* as the highest level of threat on the most recent report of antibiotic resistance threat in the United States^25^. Thus, a robust response is needed to develop new antifungals and treat such infections.

Fluconazole is the mainstay of antifungal therapy for disseminated candidiasis. Nonetheless, posaconazole is recommended as an alternative treatment option for patients who are infected with fluconazole or itraconazole-resistant *Candida*^26^. Posaconazole was discovered to be more effective than fluconazole, itraconazole, and voriconazole against *Candida* isolates based on its MIC values because of the long side chain of posaconazole, which increases its binding affinity to the target^27,28^. Therefore, in this study we evaluated the ability of the HIV protease inhibitors to synergize and potentiate the activity of posaconazole against *C. auris*.

Here, we found that posaconazole interacted synergistically with both HIV protease inhibitors (atazanavir, and saquinavir) against all 17 *C. auris* isolates. Additionally, the synergistic combinations exhibited a fungistatic activity reducing the *C. auris* CFU by 5 and 5.6-logs after 48 h as compared to the mock (negative) control in a time kill assay. Furthermore, the synergistic interaction between atazanavir/saquinavir and posaconazole has proven effective against all other clinically important *Candida* species, providing an additional clinical advantage to these combinations.

Based on these results and our prior findings, it is evident that both atazanavir and saquinavir exhibit more potent synergistic interactions when combined with itraconazole and posaconazole compared to fluconazole and voriconazole. This suggests a potential therapeutic advantage of using HIV protease inhibitors in combination with itraconazole and posaconazole, as mutations in the target gene (*ERG11*) appear to exert a lesser influence on the binding and activity of these particular antifungal agents^11,18,19^.

Biofilm formation is one of the major virulence factors of *C. auris*^29^. *C. auris* possesses high ability to form a strong adhesive biofilm on non-living structures, such as indwelling medical devices or implants^10^. Additionally, it was reported that the expression of efflux pump transporters, including both ATP-binding cassette (ABC) and Major facilitator superfamily (MFS), increased during the maturation stage of biofilm formation^3^. HIV protease inhibitors were reported to impair biofilm formation and disrupt the mature biofilm of some fungal species such as *C. albicans* and *Trichosporon*^30,31^. Therefore, we investigated the biofilm inhibition activity of atazanavir/posaconazole and saquinavir/posaconazole combinations. Both combinations significantly inhibited the *C. auris* biofilm formation.

Finally, we evaluated the synergistic combinations atazanavir/posaconazole and saquinavir/posaconazole in a *C. auris* disseminated infection mouse model. Posaconazole alone has previously demonstrated its efficacy in numerous mouse models of disseminated candidiasis^32^. However, the in vivo efficacy of posaconazole was not reported against *C. auris* before. Thus, we were encouraged to test the two combinations against mouse model of *C. auris* infection. First, we evaluated the efficacy of different doses of posaconazole to determine the optimal dose that does not result in a significant reduction of the fungal burden. We found that posaconazole’s dose of 3 mg/kg was the highest dose that did not significantly reduced *C. auris* CFU as compared to the untreated control. This dose was used in combination with either atazanavir or saquinavir to test the in vivo efficacy of the combinations against *C. auris*. Ritonavir was added to each combination as a pharmacokinetic enhancer^33,34^. Atazanavir–ritonavir and saquinavir–ritonavir did not reduce the fungal CFU, similar to previous studies^18,19^. On the other hand, the two combinations atazanavir/posaconazole and saquinavir/posaconazole, in the presence of ritonavir, significantly reduced the *C. auris* CFU burden in mice kidneys, generating 2.04 (99.1%) and 1.44-log_10_ CFU (96.4%) reduction, compared to the mock (negative) control group. Remarkably, the in vivo efficacy of the atazanavir/posaconazole combination (with 2.04 -log_10_ reduction) surpassed the in vivo efficacy of atazanavir/itraconazole combination (with 1.15-log_10_ reduction) against *C. auris*^18^. Additionally, the in vivo efficacy of the saquinavir/posaconazole combination (with 1.44-log_10_ reduction) surpassed the in vivo efficacy of saquinavir/itraconazole combination (with 0.85-log_10_ reduction) against *C. auris*^19^.

In conclusion, the atazanavir/posaconazole and saquinavir/posaconazole combinations displayed not only in vitro synergistic antifungal activity but also anti-biofilm activity against *C. auris.* Moreover, our study found that the combinations efficiently reduced *C. auris* burden in mice kidneys. Thus, atazanavir and saquinavir can be considered as promising agents for enhancing the potency of posaconazole against azole-resistant *C. auris*.

## Materials and methods

### *Candida auris* strains, reagents and chemicals

*C. auris* strains were obtained from the BEI Resources (Manassas, VA, USA), and CDC (Atlanta, GA, USA). Media and reagents were provided from the following chemical vendors: crystal violet (Acros Organics, New Jersey, USA), 3-(N-Morpholino) propane sulfonic acid (MOPS) (Fisher Bioreagents, Fairlawn, NJ, USA), phosphate-buffered saline (PBS) (Corning, Manassas, VA, USA), RPMI 1640 (Gibco, Grand, Island, NY, USA), yeast peptone dextrose (YPD) broth (Becton, Dickinson and Company, Franklin Lakes, NJ, USA), and YPD agar (DOT Scientific Inc, Burton, MI, USA). Drugs were obtained commercially as follows: atazanavir and saquinavir (Ambeed, Arlington Heights, IL, USA), chloramphenicol (Sigma-Aldrich, St. Louis, MO, USA), cyclophosphamide (Cayman Chemical, Ann Arbor, MI, USA), posaconazole (Biosynth Carbosynth, San Diego, CA, USA), and ritonavir (TCI America, Portland, OR, USA).

### Minimum inhibitory concentration and checkerboard assay

The MICs values of posaconazole, the HIV protease inhibitors (atazanavir and saquinavir) were evaluated against *C. auris* isolates following the CLSI guidelines^35^. The combinations of posaconazole with atazanavir or saquinavir were determined against 17 *C. auris* isolates using the checkerboard method, as described elsewhere^36^. Similarly, the two combinations were evaluated against other *Candida* species including *C. albicans*, *C. tropicalis*, *C. parapsilosis*, *C. krusei* and *C. glabrata*. The FICI was calculated and interpreted as follows: FICI of > 4 was classified as antagonism, FICI of > 0.5–4: indifference, and FICI of ≤ 0.5: synergism^37^. To confirm the ability of atazanavir and saquinavir to improve the activity of posaconazole against *C. auris*, we used posaconazole test strips. *C. auris* AR0390 was cultured in RPMI 1640 agar in the presence of atazanavir, saquinavir or dimethyl sulfoxide (DMSO). Next, the posaconazole test strip was added, and the agar plate was incubated at 35 °C for 24 h. The concentration at which a zone of inhibition intercepted with the posaconazole strip used as the MIC.

### Time-kill kinetics and spotting assays

A time-kill assay was used to investigate the killing kinetics of the atazanavir/posaconazole and saquinavir/posaconazole combinations against *C. auris* AR0390, as described before^17,38,39^. Briefly, exponential phase *C. auris* cells were diluted to ~ 10^4^ CFU/ml in RPMI 1640 medium and incubated with either atazanavir (16 µg/ml), saquinavir (16 µg/ml), posaconazole (0.25 µg/ml), atazanavir/posaconazole combination, or saquinavir/posaconazole combination. DMSO-treated cells were used as a negative (mock) control. Aliquots were taken at specific time points (0, 6, 12, 24, 30, 36, and 48 h), diluted and counted to determine the number of viable cells. The data are presented as the average of three independent experiments. For the spotting assay, aliquots from the time point of 24-h were plated onto YPD agar plates and incubated at 35 °C for 24 h before the plates were scanned.

### *C. auris* biofilm formation assay

The *C. auris* biofilm formation assay was performed as described previously^40–42^. To evaluate the effect of the atazanavir/posaconazole and saquinavir/posaconazole combinations in preventing biofilm formation, HIV-protease inhibitors (atazanavir and saquinavir) diluted in RPMI medium (at 16 μg/ml) with and without posaconazole (0.03 μg/ml) were added to 96-well plates containing 10^6^ CFU/ml of *C. auris* AR0390 cells, and the plates were incubated at 35 °C for 24 h. To assess the biofilm inhibition of the *C. auris* AR0390 isolate, adherent biofilms were stained for 30 min with 100 μl of 0.1% (wt/vol) crystal violet. After crystal violet was removed, the cells were washed 3 times with PBS and the plates were allowed to dry. The resultant biofilm biomasses were then quantified by dissolving the crystal violet-stained biofilms in 100 μl of ethanol before recording absorbance values (OD_600_).

### *C. auris* infection mouse model

The Virginia Tech Animal Care and Use Committee reviewed and approved the mouse study, which was conducted strictly in accordance with the National Institutes of Health Guide for the Care and Use of Laboratory Animals. The mouse studies are in compliance with the Animal Research: Reporting of In Vivo Experiments (ARRIVE) guidelines. To assess the efficacy of the two combinations atazanavir/posaconazole and saquinavir/posaconazole in vivo against *C. auris*, we used a mouse model of disseminated *C. auris* infection as reported previously^43^. First, we conducted a dose–response study for posaconazole to determine the dose that can be used in combination with either atazanavir or saquinavir. Female CD-1 mice were injected intraperitoneally (I.P.) with cyclophosphamide (200 mg/kg) and (150 mg/kg) 4 days and 1 day, respectively before infection. On the infection day, mice were injected I.P with 4.5 × 10^7^ cells/mouse of *C. auris* AR0390. Two hours later, mice were randomly distributed into 6 groups and administered different doses of posaconazole (0.25, 0.5, 1, 3, 5 mg/kg) orally once daily for two days. One day later, mice were humanely euthanized using CO_2_, and their kidneys were extracted, homogenized, serially diluted, and plated onto YPD agar supplemented with chloramphenicol. YPD plates we then incubated for 24 h at 35 °C for the CFU determination.

We determined the optimal dose of posaconazole as 3 mg/kg to be used in the following experiment. Next, we assessed the in vivo efficacy of the two combinations, with adding ritonavir as pharmacokinetic enhancer. Mice were rendered neutropenic by cyclophosphamide injection as described above. On the challenge day, mice were injected I.P. with 5.67 × 10^7^ cells/mouse of *C. auris* AR0390. Two hours after infection, mice were randomly allocated into groups and all treatments were given orally as follows: (A) Control, (B) posaconazole (3 mg/kg), (C) atazanavir–ritonavir (90–30 mg/kg), D) saquinavir–ritonavir (200–20 mg/kg), (E) posaconazole/atazanavir–ritonavir (3/90-30 mg/kg), (F) posaconazole/saquinavir–ritonavir (3/200-20 mg/kg). In these groups, posaconazole was administered once daily while atazanavir and saquinavir were administered with ritonavir twice daily for 2 days. Twelve-hours after the last dose, mice were euthanized and the *C. auris* burden in the mice kidneys was determined as described above. The data was analyzed via one-way analysis of variance (ANOVA) with post-hoc Dunnett’s test for multiple comparisons.

### Statistical analyses

The statistical tests and significance were determined and indicated in each figure legend using GraphPad Prism 8 software.

### Ethical approval

All mice experiments were approved, and performed according to the regulations of the Virginia Tech Animal Care and Use Committee.

### Supplementary Information

Supplementary Information 1.

Supplementary Figure S1.

## Data Availability

All data generated or analyzed during this study are included in the supplementary information files.
