# Supplementary material for: Enhanced antifungal activity of posaconazole against Candida auris by HIV protease inhibitors, atazanavir and saquinavir
Source: Sci Rep. 2024 Jan 18;14:1571. doi: 10.1038/s41598-024-52012-8 (PMC10796399; doi:10.1038/s41598-024-52012-8)
Supplement: Supplementary file 2 — Supplementary Figure S1. [file 41598_2024_52012_MOESM2_ESM.docx]

**Supplemental material**

**Enhanced Antifungal Activity of Posaconazole Against *Candida auris* by HIV Protease Inhibitors, Atazanavir and Saquinavir**

**
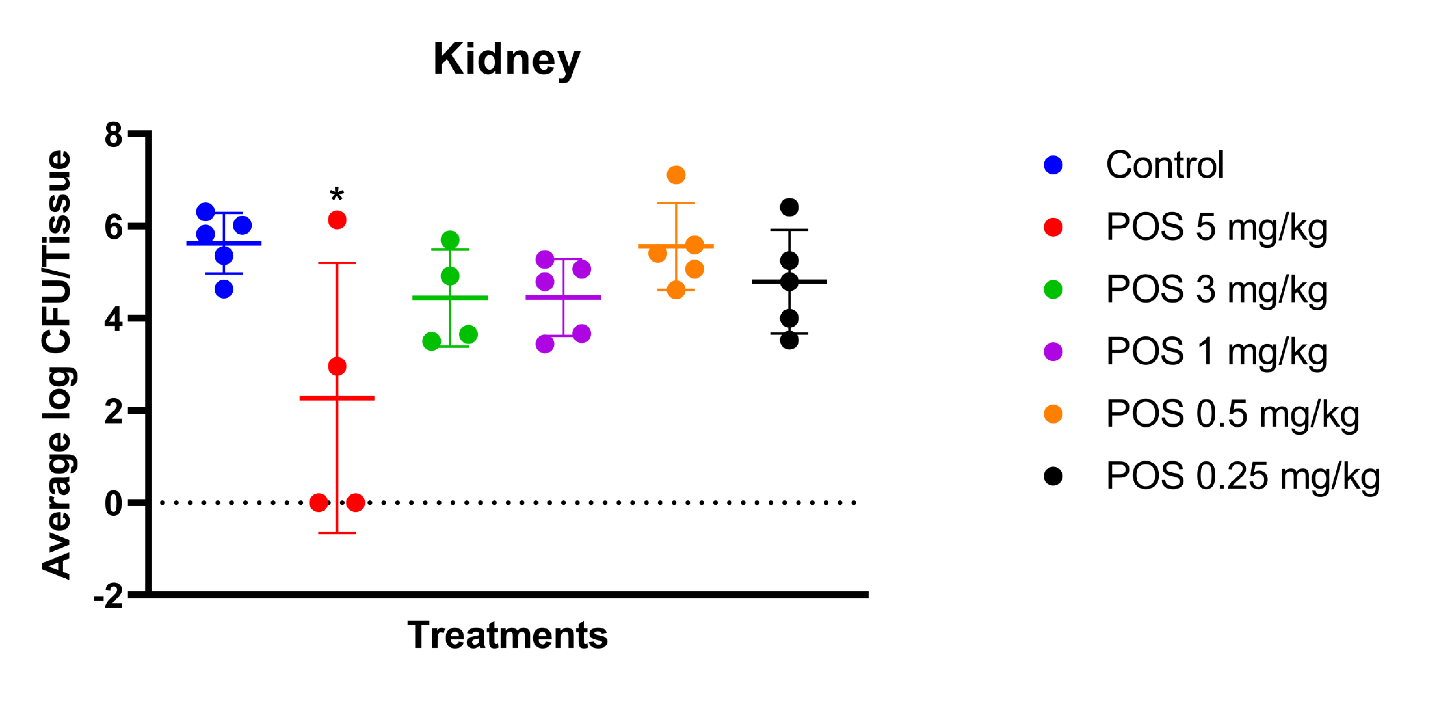
Figure S1. Optimization of the posaconazole dose against *C. auris* AR0390**. Mice were rendered neutropenic by two doses of cyclophosphamide I.P. before infection. Mice were then infected with *C. auris* AR0390. Two hours post-infection, mice were treated once daily with posaconazole (0.25, 0.5, 1, 3, and 5 mg/kg) for two days. Afterwards, mice were sacrificed, and their kidneys were removed and homogenized, serially diluted, plated on YPD agar containing chloramphenicol (100 µg/ml), and incubated for 24 hours at 35°C. The CFU data were analyzed using a one-way ANOVA with post-hoc Dunnett’s test. An asterisk (*) denotes a statistically significant difference of posaconazole-treated mice as compared to the vehicle-treated group (P ≤ 0.01).
